# Comparison of Endogenous Alpharetroviruses (ALV-like) across Galliform Species: New Distant Proviruses

**DOI:** 10.3390/microorganisms12010086

**Published:** 2023-12-31

**Authors:** Sergio Fandiño, Esperanza Gomez-Lucia, Laura Benítez, Ana Doménech

**Affiliations:** 1 Department of Animal Health, Veterinary Faculty, Complutense University of Madrid, Av. Puerta de Hierro s/n, 28040 Madrid, Spain; sergifan@ucm.es (S.F.); domenech@ucm.es (A.D.); 2 Department of Genetics, Physiology and Microbiology, Faculty of Biological Sciences, Complutense University of Madrid (UCM), C. de José Antonio Novais 12, 28040 Madrid, Spain; lbenitez@ucm.es; 3 Research Group, “Animal Viruses” of Complutense University of Madrid, 28040 Madrid, Spain

**Keywords:** Galliformes, *Alpharetrovirus*, Avian Leukosis Virus (ALV), endogenous viruses, phylogenetics, ERVs

## Abstract

The Genus *Alpharetrovirus* contains viruses pathogenic mainly for chickens, forming the Avian Sarcoma and Leukosis Virus group (ASLV). Cells of most Galliform species, besides chickens, contain genetic elements (endogenous retroviruses, ERVs) that could recombine with other alpharetroviruses or express proteins, complementing defective ASLV, which may successfully replicate and cause disease. However, they are quite unknown, and only ALV-F, from ring-necked pheasants, has been partially published. Upon scrutiny of 53 genomes of different avian species, we found *Alpharetrovirus*-like sequences only in 12 different Galliformes, including six full-length (7.4–7.6 Kbp) and 27 partial sequences. Phylogenetic studies of the regions studied (LTR, *gag*, *pol*, and *env*) consistently resulted in five almost identical clades containing the same ERVs: Clade I (presently known ASLVs); Clade II (*Callipepla* spp. ERVs); Clade IIIa (*Phasianus colchicus* ERVs); Clade IIIb (*Alectoris* spp. ERVs); and Clade IV (*Centrocercus* spp. ERVs). The low *pol* identity scores suggested that each of these Clades may be considered a different species. ORF analysis revealed that putatively encoded proteins would be very similar in length and domains to those of other alpharetroviruses and thus potentially functional. This will undoubtedly contribute to better understanding the biology of defective viruses, especially in wild Galliformes, their evolution, and the danger they may represent for other wild species and the poultry industry.

## 1. Introduction

Avian Leukosis Virus (ALV) is a globally distributed pathogen that mainly affects chickens, causing oncogenesis and immunosuppression in a fraction of infected individuals as well as a decrease in several productivity indicators [1,2]. ALV is an *Alpharetrovirus*; as such, infection is permanent among individuals and easily transmitted, especially in industrial poultry settings where chickens may be crowded. ALV has caused big economic losses to the poultry industry until efficient eradication measures were carried out in captive stocks, mainly by selecting uninfected chickens [3].

The genus *Alpharetrovirus* contains different species of avian viruses, the most relevant being ALV and Rous Sarcoma Virus (RSV), and a few defective viruses that have not been studied in depth but are mostly oncogenic. Members of the genus are also collectively known as Avian Sarcoma and Leukosis Viruses (ASLVs). However, in addition to the widely known published alpharetroviruses, there is both direct and indirect evidence of other similar viruses that exist but remain unclassified, such as Lymphoproliferative Disease Virus (LPDV), which is known to affect mainly turkeys [4,5] and whose full or nearly full-length sequences have been published in GenBank under the accession numbers KC802224 and U09568 [6], but have not been classified yet by the International Committee on Taxonomy of Viruses (ICTV).

In general, retroviruses may be classified as exogenous or endogenous. Exogenous retroviruses are transmitted between individuals, while endogenous retroviruses (ERVs) form part of the genome of the species as they are remnants of past retroviral infections of germ cells, which can be inherited by the offspring like a Mendelian gene along generations. In addition, retroviruses may become defective through genomic alterations. Non-defective alpharetroviruses have the traditional genomic organization of *gag-pro-pol-env*, while defective viruses can partially or totally lack one or more of those genes. When the viral genome is retrotranscribed to double-stranded DNA and then inserted in the host genome (provirus), it becomes flanked by two newly formed regions, the LTRs (Long Terminal Repeats). Thus, the new organization in the provirus is LTR5′*-gag-pro-pol-env*-LTR3′.

The LTR has three regions: U3 contains enhancer elements and the promoter; R is where transcription starts; and U5 contains post-translation regulatory elements [2]. LTRs and *env* are the major pathogenic determinants in retroviruses, including *Alpharetrovirus*, with relevance in the replication cycle and in tropism [7,8,9]. The *gag* ORF (*gag-pro*) encodes the structural proteins capsid (CA), matrix (MA), nucleocapsid (NC), as well as the protease (Pro). The *pol* ORF encodes the reverse transcriptase, involved in reverse transcription of the viral genome prior to insertion, and the integrase [3]. Gene *env* encodes the envelope glycoproteins, surface (gp85, SU), and transmembrane (TM). TM is important for anchoring the surface protein to the viral envelope. ALV can be distributed in subgroups with different pathogenicity according to gp85, which is the one that confers tropism through interaction with the target cell receptor. Currently, the best studied ALV subgroups are A-E and J, K. ALV A-D are practically eradicated from most breeding commercial facilities in major developed countries. ALV-J was first described in the early 1990s and expanded worldwide. ALV-K was first described in 2012 in China as an emerging subgroup and is mainly isolated in the poultry of this country, though it also occurs sporadically in other parts of the world (reviewed in [2]). ALV-E is an endogenous subgroup widely distributed in the chicken (*Gallus gallus*) genome (reviewed in [2]).

ERVs are widely distributed among all vertebrate and at least some invertebrate species [10,11]. Throughout the millions of generations of the host, ERVs may end up having different degrees of deletion or even be present as solo-LTRs as a consequence of homologous recombination between 5′ and 3′ LTRs [12]. Early evidence of ALV-like ERVs dates from as early as the 60 s [13], before the ERV phenomenon was fully understood. Initially, ERV loci were named Chicken Helper Factor (Chf), referencing their capability of acting as a helper for exogenous defective alpharetroviruses [14]. Different endogenous ALV subgroups have been identified since the 1970s, but have been poorly studied, with the exception of ALV-E, a widely distributed subgroup and the first endogenous retrovirus found in the chicken genome [15]. ALV-E is present in a low number of copies in chickens, some of which are mostly silent and some others express some proviral genes [3].

Other endogenous ALV subgroups (F, G, H, and I) have been described in Galliformes species, although they are still poorly characterized (reviewed in [2]). ALV-F was first described in 1973 from the genome of ring-necked pheasants (*Phasianus colchicus*) by the identification of exogenous viruses that were recombinants between Bryan high-titer Rous Sarcoma Virus (a defective virus that lacks the *env* gene) and an endogenous element, subsequently identified as ALV-F [16]. Other subgroups were identified following a similar approach, namely generating recombinant viruses between defective viruses combined with the putative *env* homolog from ERVs from other species and analyzing the inhibition of replication (interference properties) with the different ALV subgroups. ALV-G was detected in 1974 in golden pheasant (*Chrysolopus pictus*) [17], ALV-H was identified in Hungarian partridge (*Perdix perdix*) [18] and ALV-I was identified in Gambel’s quail (*Callipepla gambelii*) [19]. Each of these recombinant viruses showed different interference properties and host ranges; thus, they were identified as new subgroups. Research at the time discussed that ALV-F and ALV-G were not related to ALV and that they belonged to different viral species or different species complexes [20]. In addition to these subgroups F to I, Dimchef et al. [21] reported the presence of ALV-like loci in 26 species of Galliformes species using partial *gag* sequences. Later, one of those ERVs was fully sequenced, the Tetraonine Endogenous RetroVirus (TERV) from the ruffed grouse, *Bonasa umbellus* [22].

Whether these partially characterized endogenous ALV or ALV-related viruses could play any role in the pathogenesis of different processes is still controversial. It is known that, despite rarely being pathogenic directly, ALV-E is related to several pathogenic processes, such as lymphomas in the presence of other oncogenic viruses [23], ovarian adenocarcinomas [24], or even a decrease in productive traits [25,26]. ALV-E can induce immune tolerance in chicks expressing shared proteins among all ALV; but it may also benefit immunity against exogenous ALVs by different mechanisms, such as receptor interference [27,28,29], (reviewed in [2]).

Another aspect in which ERVs may play an important role is in the emergence of new subgroups through recombination. In the last decades, new viral subgroups such as ALV-J and ALV-K have arisen in chickens [30,31]. ALV-J is highly pathogenic and has a SU domain with a different origin than other ALVs, seemingly obtained through recombination with a different family of ERVs, called EAV-HP [32]. Through the last decade, different recombinant strains of ALV have been isolated [33,34], highlighting the danger of recombination between subgroups or between exogenous alpharetroviruses and different ERVs, including some lesser-known ones like ALV-F/G/H/I or others yet to be identified [2]. These include ERVs from species that have been partially sequenced [21] as well as those that are thought to exist because cells of some species have helper activity for defective RSV [20]. Therefore, it is of great interest to better understand the presence of endogenous ALVs or ALVs-like in different Galliformes and in species other than chickens, which could also help to better elucidate the host range of these viruses and their relationship with the diversity and geographical expansion of different species of birds.

Recent advances in Whole Genome Sequencing (WGS) have made possible the publication of the genomes of other Galliformes and several species of other avian orders in recent years, allowing the search for those proviruses. Consequently, the aim of the present work has been to study the presence of ALV-related proviruses in many Galliformes species that had previously been reported to exhibit helper activity or contain homologous sequences to ALV using these published genomes [20,21].

## 2. Materials and Methods

### 2.1. Subject Genomes Analyzed

The publicly available genomes of 53 species from 11 avian orders were examined to locate potential ERV sequences. This included, amongst others, Anseriformes, Passeriformes, Psittaciformes, and Galliformes (Table S1). ERVs were located only in twelve species from two families of Galliformes, Odontophoridae and Phasianidae (Table S1), which were analyzed in detail. Our choice to survey such diverse taxa was prompted by the fact that there is non-conclusive evidence of the presence of ASLV infections in distant species such as parakeets [35], ostriches [36], house sparrows [37], ducks [38,39], or other small Passeriformes [38].

### 2.2. Data Mining

**Figure 1:**
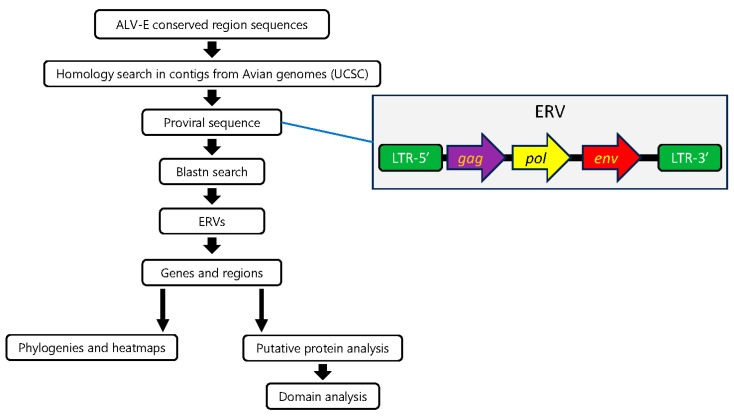
Workflow for finding ALV-like ERVs. Conserved regions from ALV-E were used to search in different avian order genomes. The longest proviral element from each species with the most recognizable genes was used in the Blastn search to find other ERVs in the same species. For each ERV collected this way, genes and regions were separated and used in a joint analysis with the equivalent regions from ERVs from other species.

The workflow is shown in Figure 1. ALV-E proviral sequence (*Gallus gallus*, AY013303) was used to locate similar sequences in the publicly available genomes in the UCSC Genome Browser (University of California Santa Cruz, http://genome.ucsc.edu (accessed on 6 June 2023).). For this search, we compared the subject genomes with four regions in the AY013303 genome: the LTR sequence, U5, 1.5 Kbp from the start or end of the *gag* ORF, and the TM region. Using this approach, we obtained several 7–8 Kbp or smaller ERVs for each species, usually flanked by both LTRs, which kept most genes in a recognizable state, although for species with a low number of ERVs (*Colinus virginianus*, *Lagopus muta*, *Tympanuchus cupido*), we relaxed the criteria to be able to include them in the analysis. If the ALV-E sequence search yielded no results, we then used the same approach using the *Alectoris rufa* provirus sequences from CAMZON010000074 (LTR, U5, and 1.5 Kbp from the start or end of the *gag* ORF) as a query in the search. The *Alectoris rufa* ERV was chosen because it had a high degree of gene conservation and, consequently, constituted a good ERV to compare with. In addition, we expected that the rest of the ERVs could be potentially divergent from ALV-E, even in conserved regions, because of the different selective pressures between wildlife and domestic avian species, a difference that is known to have caused an enrichment in ALV-E insertions close to protein-coding genes [29]. All newly found ERV sequences (whose coded name is shown in Table S2) were analyzed using RepeatMasker (https://www.repeatmasker.org/cgi-bin/WEBRepeatMasker (accessed on 20 June 2023).) which detects sequences related to retroviral elements. If the search in UCSC retrieved a poor-quality proviral sequence (i.e., a sequence with a high percentage of N), we used Blastn to compare it to its species’ WGS/genome and downloaded the resultant sequence along with 7–8 Kbp. Once annotated, this ERV was used again in Blastn to find other ERVs in the same species. Automated software such as RetroTector v.1.0.1 [40] was not used because of its deficient efficiency in finding ancient ERVs correctly [41]. The reference sequences used for currently known ALV subgroups were selected because they have been widely used in other studies.

### 2.3. Sequence Analysis

The first stage included mapping LTRs, *gag*, *pol*, and *env* in the *Alectoris rufa* CAMZON010000074 provirus (Alectoris-rufa1) by searching regions that displayed homology with ALV-E in the software Vector NTI v11.5.2 (https://www.thermofisher.com/es/es/home/life-science/cloning/vector-nti-software/ (accessed on 25 June 2023) [42]). In the other ERVs, the start and end of each gene were mapped by comparison with the first 20–30 bp from ALV-E AY013303 or from the Alectoris-rufa1 provirus. A matching rate of more than 60–70% was considered a match. When this method did not allow resolution, we aligned the regions of the subject provirus with the ALV-E equivalent region using the Muscle algorithm in MegaX [43]. Each of the regions present in the provirus (LTR5′, *gag, pol,* and *env*) was analyzed individually. The LTR3′ was not used at this stage, as in most cases, both LTRs were almost identical.

We used the part of the CoDing Sequence (CDS) that encodes each of the mature proteins for *gag* and *pol* (i.e., CA, MA, etc.), but *env* phylogeny was created using the whole *env* CDS. *env* CDS is known to include splicing in ALV and RSV; thus, we checked our sequences with the Netgene2 server (https://services.healthtech.dtu.dk/services/NetGene2–2.42/ (accessed on 26 July 2023) [44]). We also included other sequences from the ASLV group: Avian carcinoma Mill Hill virus, Avian sarcoma CT10 virus, Fujinami sarcoma virus, UR2 sarcoma virus, Y73 Sarcoma virus, *Bonasa umbellus* ERV (TERV), and Lymphoproliferative disease virus (LPDV), which is known to be similar to *Alpharetrovirus* [4] and a full sequence is available in KC802224 in GenBank [6]. For *env* analysis, we also included a sequence from each ALV subgroup A, B, C, D, E, J, and K. The complete list of GenBank Accession Numbers is shown in Table S3. A separate analysis was conducted just for the U3 and the R-U5 regions of LTR because R-U5 is usually very conserved, even among proviruses from different hosts, and U3 shows extreme variability.

### 2.4. Phylogenetic Analysis

Analysis was performed using the Maximum Likelihood method in MegaX v.10.2.6 (https://www.megasoftware.net/download_form (accessed on 30 July 2023) with 1000 bootstrap replicates and the substitution model appropriate for each alignment. Alignments for the trees were performed using Muscle v.5 (https://github.com/rcedgar/muscle (accessed on 30 July 2023)) [45]. The trees were edited using the Interactive Tree of Life (ITOL [46]) website (https://itol.embl.de/ (accessed on 14 September 2023).) In parallel, a heatmap matrix was created for each alignment using the sequence demarcation tool (SDT1.2) developed by Brejnev et al. [47]. A molecular clock approach was taken to study the *Phasianus colchicus* and *Alectoris magna* proviruses, which were shown to have the highest divergence between LTRs when the LTRs of other ERVs mapped only had 1–2 bp differences. The classical T = (D/R)/2 for LTR comparison [48] was used, where T is insertion time in millions of years, D is the number of differences per nucleotide per site, and R is the nucleotide substitution rate. We used the genomic substitution rate per site per year estimated for landbirds (2.2 × 10^−3^, [49]). To compare the phylogenies with the geographic distribution of hosts, we used the distribution maps for each species of interest from Bird Life International (http://datazone.birdlife.org (accessed on 21 September 2023)) to build a matrix showing habitat overlapping or no overlapping for each pair of species. The matrix was used to elaborate a Venn diagram.

### 2.5. Protein Analysis

The protein sequences were analyzed by ORFinder (https://www.ncbi.nlm.nih.gov/orffinder/ (accessed on 30 September 2023).) Sequences with premature stop codons were considered dysfunctional, and protein sequences that span until near the equivalent stop codon in ALV-E were considered to be possibly functional (putative proteins) because it is known that many ALV-E retain functional ORFs and are even capable of producing infective viral particles [3]. Domains have been analyzed using NCBI’s Conserved Domain Database (CDD) v3.20 [50] (https://www.ncbi.nlm.nih.gov/Structure/cdd/wrpsb.cgi (accessed on 5 October 2023)) only in the potentially functional proteins.

## 3. Results

We explored the existence of ALV-like sequences in the genomes of 53 avian species of 11 different orders, but they were only found in Galliformes. Of these, most species yielded no successful ERV results (Table S1), but several full-length ALV-like proviruses were located in six of the studied genomes: *Alectoris rufa*, *Alectoris magna*, *Callipepla californica*, *Centrocercus urophasianus*, *Centrocercus minimus*, and *Phasianus colchicus* (Table 1). ERVs lacking some regions were recovered from another six species: *Callipepla squamata*, *Colinus virginianus*, *Lagopus leucura*, *Lagopus muta*, *Tympanuchus cupido,* and *Tympanuchus pallidicinctus*. Some ERVs with smaller sizes or solo LTRs were also found but not analyzed since they were considered degenerated. All full proviruses ranged between 7.4 and 7.7 Kbp, the same sizes that are expected for ALV-E and very close to exogenous ALV sizes (7.7 Kbp). Partial sequences or proviruses that lacked genes were found instead. However, when performing a Blastn search, complete ERVs appeared despite being absent in the UCSC browser. Genes and regions were annotated as described in Materials and Methods by comparison with ALV-E (AY013303) and *Alectoris rufa* provirus from the WGS contig with accession number CAMZON010000074. For species with a low number of ERVs (*Colinus virginianus*, *Lagopus muta*, and *Tympanuchus cupido*), we relaxed the criteria to be able to include them in the analysis. *Colinus virginanus* proviral sequences had very low quality, and only the full LTR3′ sequence could be recovered from the contig with accession number VONY02000001; due to the small differences in the other proviruses between the LTR5′ and the LTR3′, it was added to the analysis with the rest of the LTR5′ sequences. *Tympanuchus cupido* WGS (MOXI01000063) was also low quality in certain regions, which made it impossible to retrieve a LTR sequence, although a characteristic U3 pattern was observed. A summary of hosts and proviral features is presented in Table 1.

### 3.1. Genetic and Protein Analyses

The phylogenetic analysis of all regions of the retroviral genomes gave similar results. Briefly, different clades could be identified, containing the same ERVs, regardless of the region studied. These were the following: subtypes of currently known exogenous ALV, along with the endogenous ALV-E and RSV ERVs (Clade I); *Callipepla* spp. ERVs (Clade II); *Phasianus colchicus* ERVs (Clade IIIa); *Alectoris* spp. ERVs (Clade IIIb); and *Centrocercus* spp. ERVs (Clade IV). Clades IIIa and IIIb were always quite close to each other. Depending on the region studied, some other ERVs had a different phylogenetic relationship. For example, in the LTR5′ phylogeny, ALV-E was more distant from Clade I than in the other regions (Figure S1). In the next sections, only differences will be highlighted.

#### 3.1.1. LTR

Both LTRs from the same provirus showed 100% identity in most cases, with the notable exceptions of *Alectoris rufa, Alectoris magna,* and *Phasianus colchicus* (Table S4). In *Alectoris rufa*, where only one pair of LTRs were analyzed (N = 1, Alectoris-rufa1), LTRs presented only one difference in 251 bp (99.6% identity). In *Alectoris magna* (N = 4), identity ranged between 99.2 and 100%. *Phasianus colchicus* (N = 6) showed a minimum of 98.8% identity for Phasianus-colchicus1-3, while the others ranged from 99.6% to 100% identity. Due to the low number of samples, we cannot conclude with certainty when the first insertions took place. We used the *Phasianus colchicus* and *Alectoris magna* proviruses with the most divergent LTRs (Phasianus-colchicus1-3 and Alectoris-magna2-1) to estimate how long ago those ERVs had been inserted in that genomic location using molecular clock. We detected 3 and 2 differences in 251 base pairs, respectively, which amount to an overall nucleotide divergence (D) of 0.0120 and 0.0080, respectively. Thus, the estimated insertion times for these proviruses are 2.73 and 1.82 million years ago, respectively. Blastn analysis using Phasianus-colchicus1 showed that the *Phasianus* genome had traces of previous ERV insertions in the form of a high number of LTR5′-LTR3′ sequences, resultant of a much longer evolutionary history since ERV endogenization. This is something that was also observed when using Blastn with ERVs from the other hosts and is already known from the literature [51].

Alignment of the LTRs showed that U3 was highly divergent amongst the ERVs analyzed, while R and U5 were well conserved (Figure S2). The LTR5′ phylogenetic tree (Figure S1) showed the different clades mentioned above with some variations. *Lagopus leucura* and *Colinus virginianus* ERVs (that we were only able to compare in the LTR region) did not cluster with other ERVs; Clade I contained, besides the LTR sequences from exogenous ALV-A and RSV mentioned above, the ERVs from *Tympanuchus pallidicinctus* and *Lagopus muta* (which in the *env* phylogenic tree were assigned to Clade IV). As mentioned above, ALV-E did not classify in Clade I but was outside (Figure S1).

#### 3.1.2. *gag* Genes

**Figure 2:**
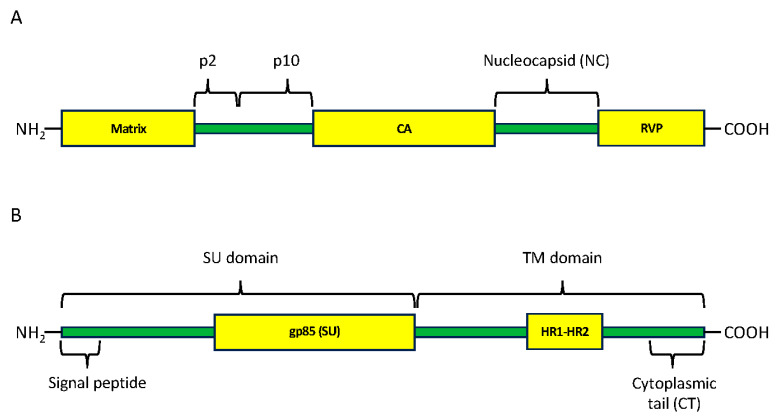
Domains and regions present in Gag polyprotein (**A**) and in Env protein (**B**). Yellow boxes are hits present in the CDD database. Green bars are sequences present in GenBank but that did not identify hits in the CDD database. CA, capsid protein; RVP, RetroViral Protease-like domain; SU surface; TM, transmembrane; HR1-HR2, heptad repeat regions 1 and 2. The signal peptide is involved in protein maturation and is cleaved at the 3′ end [52]. We have included the signal peptide when making our phylogenies. gp85 (SU) CDD hit only detects part of the SU domain.

The analysis of the aminoacid sequences of Gag putative polyproteins showed that 16 sequences (Table S5) were considered to be intact and had a stop codon close to the equivalent position in ALV-E. All Gag polyproteins ranged from 701 aa to 736 aa (Table S5). Amino acid sequences were homologous except in part of the matrix (MA) and in the poorly characterized p2 and p10 regions (probably associated with pathogenicity), where there were indels specific to some taxa. The MA domain was detected in all amino acid sequences, along with the Gag p24 domain, which encodes the capside (CA) protein domain. Lastly, all sequences contained a retroviral protease-like (RVP) domain necessary to cleave polyproteins, which is present in retroviruses, including RSV and ALV (Figure 2A).

The ERV *gag* ORFs phylogenetic tree followed a similar distribution to the general one mentioned above. However, Clade IIIb (*Alectoris* spp. ERVs) seemed to be closer to Clade IV (*Centrocercus* spp. ERVs) than to Clade IIIa (*Phasianus colchicus* ERVs), contrary to other regions (Figure S3).

#### 3.1.3. pol Genes

A domain search in the CDD was performed with Pol polyprotein segments. *pol* may encode putative proteins in 19 ERVs, which lacked early stop codons. Those ERVs corresponded to *Alectoris rufa*, *A*. *magna*, *Callipepla californica*, *C. squamata*, *Centrocercus urophasianus*, and *Phasianus colchicus*. Putative protein sizes were either 895 or 901 amino acids long (Table S5). No particular region seemed to accumulate amino acid differences or indels. CDD searches found exactly the same identical domains and motifs in all 19 sequences: a retrotranscriptase (RT) domain, an RNAse-H domain (retroviral RNAse), and an integrase domain (IN). Gene *pol* was deleted in *Lagopus* and *Tympanuchus* ERVs (Table 1). In the rest of the ERVs, it was either a potentially functional ORF or a pseudogene.

**Figure 3:**
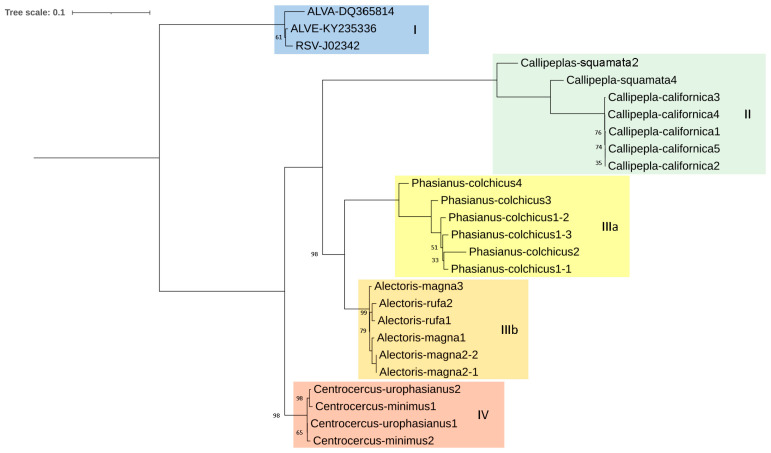
Maximum likelihood tree based on *pol* CDS sequences (2708 bp) of several proviruses detected in seven species of Galliformes using the K2 + G+ I method (bootstrap value = 1000 replicates). ALVA: Avian Leukosis Virus subgroup A; ALVE: Avian Leukosis Virus subgroup E; RSV: Rous Sarcoma Virus. Only bootstrap values lower than 100% are displayed. I, II, IIIa, IIIb and IV denotate the proposed clades.

**Figure 4:**
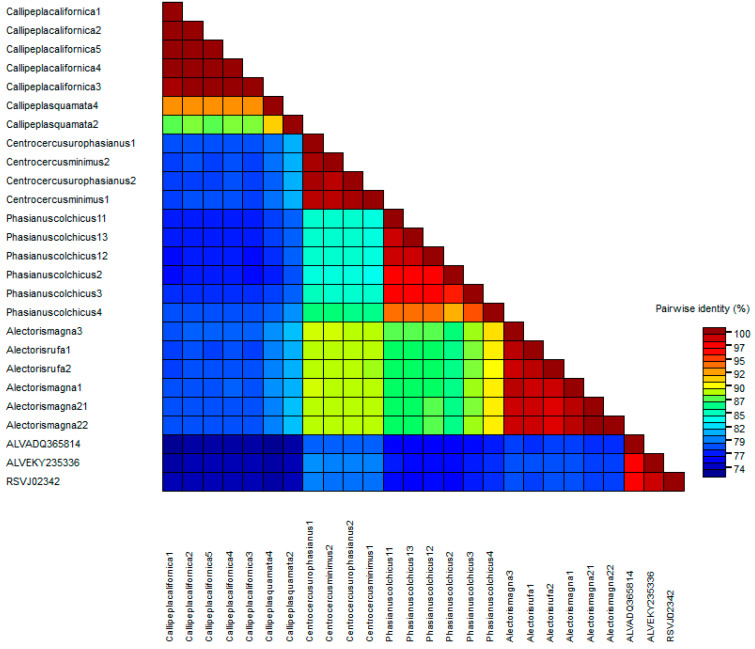
Heatmap matrix showing the identity percentages of *pol* CDS sequences using the demarcation tool 1.2. Note that orange and blue colors represent an identity lower than 95%.

The *pol* phylogenetic reconstruction (Figure 3) followed the general scheme mentioned before. It is worth noting that identities between sequences were mostly higher than 75% (Figure 4). An identical tree was obtained by computing the retrotranscriptase (RT) region of *pol*, emphasizing that the whole *pol* gene and RT region are equally conserved.

#### 3.1.4. *env* Genes

*env*-encoded putative polyprotein sequences had no early stop codons in 15 ERVs. Those ERVs corresponded to *Alectoris rufa*, *A*. *magna*, *Callipepla californica*, *Centrocercus urophasianus*, *Phasianus colchicus*, and *Lagopus muta*. In these, the putative polyprotein size ranged from 568 to 628 amino acids (Table S5). CDD domain search identified a part of the gp85 (SU) domain of 57 amino acids in *Callipepla californica* and 214–265 amino acids in the rest of the host species (Figure 2B). Amino acid sequences were homologous between all of them only until 40 amino acids downstream, approximately at the signal peptide; downstream, they became diverse, including many indel regions, especially in *Callipepla californica*. Regarding variable regions (vr) vr1, vr2, vr3, and hypervariable regions (hr) hr1 and hr2, the relevant regions of Env for host range and subgroup [2], we found that the vr1 equivalent region was only present in *Phasianus colchicus* ERVs, while vr2 was highly conserved in most cases and vr3 was not conserved among different ALV-like ERVs, though *Phasianus colchicus* and *Alectoris* vr3 kept some ressemblance. hr1 and hr2 sequences were not conserved among ERV sequences. The Heptad Repeat 1-Heptad Repeat 2 region (HR1-HR2), which is responsible for the transmembrane part of the TM domain, was found in all sequences. Their amino acid sequences were significantly different from those of ALV-A and ALV-E. The TM domain was highly conserved. The C-terminal end of Env, which corresponds with the cytoplasmatic tail (CT) domain, was highly heterogeneous. Identified ERV sequences had two different types of CT: *Alectoris rufa*, *A. magna*, *Centrocercus urophasianus*, *Phasianus colchicus,* and *Lagopus muta* had the same length as ALV-A and E subtypes (25 aa), but *Callipepla californica* was eight amino acids shorter, with the same length as ALV-J CT.

**Figure 5:**
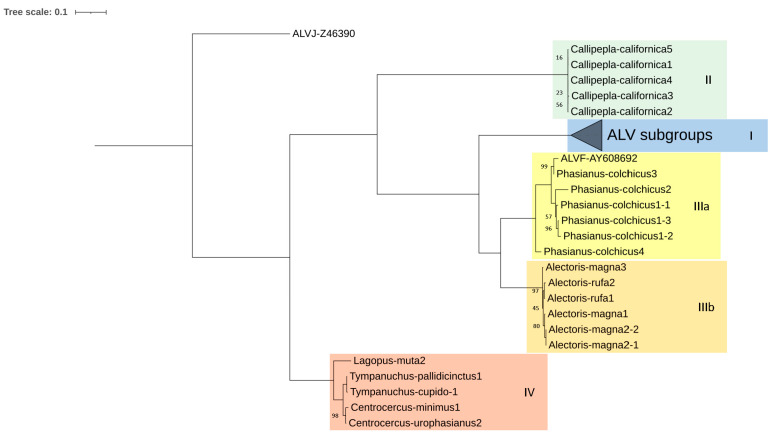
Maximum likelihood tree based on *env* CDS DNA sequences (1887 bp) of several proviruses detected in seven species of Galliformes using the K2 + G+ I method (bootstrap value = 1000 replicates). I, II, IIIa, IIIb and IV denotate the proposed clades. Clade I contains ALV-A, B, E, K, and RSV-C, D sequences, and it has been collapsed for a better understanding of the figure. Only bootstrap values lower than 100% are displayed.

In the *env* phylogenetic tree (Figure 5), *Lagopus* and *Tympanuchus* sequences, which could not be included in the *gag* and *pol* trees, were grouped together with *Centrocercus* in Clade IV. The ALV-J *env* sequence, which originated due to recombination with EAV-HP ERV [32], fell outside the remaining Clades.

### 3.2. Relationships between ERVs: Phylogenetic Inference and Geographic Distribution of Their Host Species

**Figure 6:**
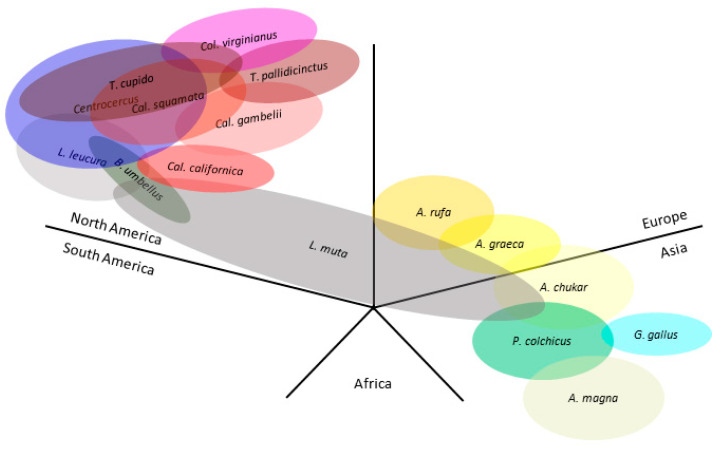
Schematic representation of the native distribution map of hosts that carry the studied proviruses. Data from Bird Life International (https://www.birdlife.org/ (accessed on 21 September 2023)). Each section corresponds to a geographical region. Other relevant species have been included: *Bonasa umbellus*, *Callipepla gambelii*, *Alectoris graeca*, *A. chukar*, and *Gallus gallus*. *A., Alectoris*; *B., Bonasa*; *Cal., Callipepla*; *Col., Colinus*; *G., Gallus*; *L., Lagopus*; *P., Phasianus*; *T., Tympanuchus*. Oval overlapping is indicative of native habitat overlapping between species. Close distribution limits between species of less than 100 km have been considered overlapping since they may mean a possibility of interspecies contact. Oval size or degree of overlapping between ovals does not correspond with quantitative values.

As the clades formed by the phylogenetic analyses were mostly preserved in the different regions, we studied the geographical ranges of each host species and of related species of interest (Figure 6). The North American species were highly interconnected; they constitute the hosts of the ERVs in Clades II and IV, along with others, such as *Colinus virginianus* or *Tympanuchus* spp. On the other hand, the Eurasian species are the hosts of the ERVs of Clades IIIa (*Phasianus colchicus*) and IIIb (*Alectoris* spp.), which are grouped close together in the phylogenetic trees. Figure 6 includes other relevant species: *Bonasa umbellus*, carrier of TERV; *Callipepla gambelii*, carrier of ALV-I; *Alectoris graeca* and *A. chukar*, which form a superespecies with *A. rufa* and genes may jump from one to another [53]; and *Gallus gallus*, host of ALV-E, which emerged in the Asian ancestor red jungle fowl. *Lagopus muta* has a high and mostly continuous distribution range and may connect the Eurasian and North American species, possibly playing a role in the distribution of ALV-like retroviruses. South American, African, and Oceanian Galliformes species are currently absent in GenBank genomes.

## 4. Discussion

Retroviruses have been infecting animals for millions of years. Sometimes they have succeeded in infecting germ cells, spermatozoids, and oocytes, and they remain in the genome of the species as ERVs. Integrations with negative consequences must have been eliminated, but many of the integrations must have positive or neutral outcomes because up to 10% of the genome of vertebrates is of retroviral origin [54]. Thus, it is of undeniable interest to learn about these sequences in the different species. In addition, ERVs have a high potential for recombining with exogenous retroviruses, with the ensuing risk of forming new viruses of unknown pathogenicity [2,27]. In this study, we have analyzed the phylogenetic relationships between ALV-like ERV sequences and putative proteins encoded in different Galliformes.

In an effort to uncover the non-studied ALV-like ERVs, we attempted to search the genomes of different avian orders published in the UCSC database, one of the most complete, fast, and easy-to-use databases. Dimcheff et al. [21] reported the presence of ALV-like loci in 26 species of Galliformes using partial *gag* sequences. These included most of the species for which we have obtained proviral sequences: *Phasianus colchicus*, *Centrocercus urophasianus*, *Lagopus leucura*, *Tympanuchus pallidicinctus*, *Colinus virginianus,* and *Callipepla californica*. Genomes from other Galliformes studied by Dimchef et al. [21], such as *Numida mealeagris,* did not give results in the search in the UCSC database, while others, such as *Colinus virginianus* and *Tympanuchus cupido*, offered poor quality contigs. To obtain these sequences, we consulted the GenBank database. It is worth noting that new ALV-like sequences were not detected in the genomes of other avian orders (Table S1), making it an exclusive Galliformes character. Unfortunately, the genomes of African, Oceanian, and South American Galliformes, with which it would have been interesting to compare, are missing from the databases at the time of this study.

In the phylogenetic analyses, we chose to use DNA sequences instead of amino acid sequences because ORFs from ERVs often have premature stop codons due to indels or substitutions, which make them difficult to compare. Nevertheless, an early stop does not preclude the risk of recombination with different exogenous or endogenous retroviral elements. However, ERV genes can be preserved with an intact open reading frame (ORF), allowing the expression of functional viral proteins and infective virions like in some ALV-E loci [3].

Even though the sequences were from two different families (Odontophoridae and Phasianidae) and three subfamilies from Phasianidae (Perdicinae, Tetraoninae, and Phasianinae), the alignments showed high similarities among most sequences for the major genes. However, there were slight differences in the way they were grouped in the phylogenetic trees. The fact that there are discrepancies among the various phylogenies of the different proviral genes and that they can offer different phylogenetic relationships may not be as exceptional as it may seem [55]. There are multiple causes of this phenomenon, but evolutionary rates due to different selective pressures, recombination, and mutations may be involved and cause the point differences between the phylogenetic inference results of the different ALV-like ERV genes. In addition, the results of the phylogenetic analysis may be biased because they do not include all ERV genes from all species analyzed.

In our study, the spectrum spanned from the high variability of U3 in the LTR5′ to the conserved *pol* sequences. U3 was very different in the various Galliformes (Figure S2). Since U3 is responsible for transcription regulation, it is subject to selective pressures from environmental factors that affect the host and even require the acquisition or loss of transcription binding sites (TBS) (Fandiño et al., manuscript in preparation), which may also cause an adaptive selection for tissue tropism [56,57]. On the contrary, the R-U5 region was highly conserved, which might be related to the relevance of the post-transcriptional processes U5 regulates [58].

The other end of the spectrum is represented by the *pol* gene, which is very conserved both in the proviral DNA and the predicted amino acid sequences and length of the encoded proteins. Gene *pol*, and more specifically, its RT domain, has been suggested by the ICTV as the demarcation criteria between retroviral genera [59]. Though ICTV does not give a specific value to consider a virus or set of viruses as a different species within the *Alpharetrovirus* genus, the analysis of the *pol* sequences assigned to the ALV and RSV species in GenBank suggests that the cut-off value may be 95% identity. The *pol* identities of the ERVs analyzed compared with chicken ASLVs were below 95% (Figure 4). Thus, we suggest that most of them belong to different “ERV species”. The *pol* phylogenetic tree (Figure 3) provides a clear picture of five different clades in which the new *Alphavirus*-like ERVs may be grouped: chicken ASLV already recognized by the ICTV (Clade I), North American quails (Clade II), Eurasian pheasants (Clade IIIa), Eurasian partridges (Clade IIIb), and North American grouse (Clade IV). All CDS sequences kept a similar level of identity between each other, except *Callipepla californica* and chicken ASLVs, which were highly different from clades IIIa, IIIb, and IV.

Our results of the classification in these Clades are also supported by the distribution of species and the moments of speciation predicted by the published phylogenies of Galliformes [60,61]. The five species in the North American grouse clade (Clade IV) are phylogenetically close. If all the ERVs in this study were related, insertion is estimated to have happened 39.9 million years ago [62,63], or if only Phasianidae are considered and *Callipepla* and *Colinus* are excluded because they belong to a different phylogenetic branch (Family Odontophoridae), that estimate would be 35.2 to 42 million years ago [63,64,65]. This dating implies that an ancient retrovirus became endogenized in a common ancestor of the present Galliformes and underwent genetic drift with high levels of divergence in ERV genes among different families and subfamilies. However, as most ORFs in these host species maintain integrity and are equivalent to those in ALV-E (Table S5), being putatively functional, this hypothesis seems unlikely. Thus, it is more conceivable that several endogenization events took place. In the case of *Phasianus* spp. and *Alectoris* spp., though they have been grouped close as Clades IIIa and IIIb, respectively, their analysis of the phylogeny suggests that the ERVs they harbor may be too distant to be considered to belong to a single Clade. It may have happened that they were infected by a related virus or were cross-infected as their habitats overlapped (Figure 6).

To better clarify the dating for the endogenization process, the differences between the LTR5′ and LTR3′ may be used to establish when the retroviruses integrated into the germline, as originally both must have been identical but went through mutations over the generations. With this technique, it has been shown that the most ancient ERVs subjected to LTR dating became part of the germline even before the Ordovician period, maybe in the Paleozoic era 460–550 million years ago [66]. Dating of *Phasianus colchicus* and *Alectoris* ERVs using LTR identity showed that ALV-like ERVs, putatively *Alpharetrovirus*-like, already existed 2.7 million years ago. Thus, on a geologic scale, the endogenization process of these ERVs is quite recent, at least in those we have been able to study because they had both LTRs. In fact, some endogenous ALV-like viruses may end up having different degrees of deletion or even be present as solo-LTRs as a consequence of homologous recombination between 5′ and 3′ LTRs [12] and may disappear with time, becoming indistinguishable from the rest of the genome. Due to the low number of samples, we cannot conclude with certainty when the first insertions took place. In addition to the initial integration, most of the ERVs in the different genomes are probably the result of reintegrations in different parts of the genome [67]. This may explain why Blastn analysis of the ERVs in *Phasianus* and in the rest of the host genomes showed a high degree of partially deleted ERVs and LTR5′-LTR3′ traces of ERV insertions, consistent with a much longer evolutionary history since ERV endogenization than the one we can estimate through LTR identity [67].

Though *env* was the most divergent gene, most ERVs showed high identity at the start of SU and at the TM domains of these proteins. Such regions are important for maturation (signal peptide) and for the adequate presence of surface proteins in the virion [52]. Many *env* ORFs seemed to have no stop codons, be of a similar length to those of ALV-E, and thus be potentially functional. If the *env* region recombines with exogenous alpharetroviruses, it may encode an operating protein, which may change the tropism of the virus. In fact, subgroups F–I were identified by recombination and interference properties. As seen in Figure 5, *env* ALV sequences are monophyletic with the exception of ALV-J, which is coherent with previous knowledge [68]. As mentioned before, ALV-J *env* is a recombinant between ALV *env* and EAV-HP *env* [3,32]. Domains found in all *env* amino acid sequences were typically ALV-related *env* motifs: gp85 (SU) and HR1-HR2 heptad repeat domains (in TM), which are the backbone of gamma-type Env proteins [52]. The amino acid sequences kept low identity among all ERVs and with the known ALV species (Figure S4), especially in the SU region. This may mean that these gp85 sequences are possibly new *Alpharetrovirus* subgroups with different interference patterns and may have different target receptors than chicken ALV subgroups, which so far are Tva (ALV-A and ALV-K), Tvb (ALV-B, ALV-D, and ALV-E), Tvc (ALV-C), and chNHE1 (ALV-J) [2]. This was especially evident in the amino acid sequences of *Callipepla californica*. Heterogeneity was quite noticeable in the variable regions (vr), which are the main domains that interact with the cell receptor by being part of the receptor binding domain [52], supporting the potential use of different cell receptors. The final part of *env*, which encodes the cytoplasmic tail (CT), was highly variable in our sequences, both in length and in sequence. In other retroviruses, such as HIV, the CT has been proven to be required for replication in certain cell types [69]. The tail is involved in anterograde trafficking, retrograde trafficking, membrane localization, Env packaging into particles, interaction with signaling pathways, or regulation of Env conformation, among other functions; however, most of these assertions come from lentiviruses with a significantly longer CT tail [52,70], which may mean some of these functions may not occur in shorter CT tails such as the ALV tail, which is approximately 25 aa long [69], as most of the ERVs studied, and 17 aa in *Callipepla californica* (as in ALV-J). Since the functions listed above are host- and Env-specific, it makes sense that the CT tail is highly variable among ERVs from different species.

Subgroups ALV-F through I have not received as much attention as the other subgroups. One of the reasons may be that they seem to be laboratory products, obtained artificially through recombination and phenotype mixing. To the best of our knowledge, AY608692 is the only public ALV-F sequence, which includes only the ALV-F *env* sequence. The fact that we have also found ALV-like proviruses in the same host, *Phasianus colchicus*, and the high identity they share with AY608692 suggests we have found complete ALV-F sequences, a “subgroup” whose full sequence was previously unknown. Having a potentially functional *env* gene also supports that this ERV was initially identified by phenotype mixing when cells were cocultured with a defective virus [17]. However, due to having a *pol* identity lower than 85% with known *Alpharetrovirus* (Figure 4), we suggest that it be treated not as an ALV *sensu stricto* (ALV-like viruses found in birds), but as a distant relative, which was termed Ring-necked Pheasant Virus (RPV) in the original papers from the 1970s [17,68]. The same treatment would be applicable to the remaining clades of ERVs studied by us.

As we mentioned before, ALV-I was isolated as a recombinant virus from *Callipepla gambelii* [19]. There is not a currently published *Callipepla gambelii* genome; thus, we have studied *Callipepla californica* and *C. squamata* contigs. Since the three species belong to the same genus, we may consider that *Callipepla californica* ERVs may have a similar sequence to ALV-I. C. squamata ERVs, which have a high degree of deletions, are different from *C. californica* ones, especially in the *pol* and *gag* sequences, and that none of the *C. squamata* ERVs had an apparently functional *env* gene. This might be explained by the fact that *C. squamata* belongs to a different branch of the genus [71] and separated earlier from the rest with *C. douglasii*. The distance between *C. gambelii* and *C. californica* is estimated to be between 1.1 and 2.5 million years ago [63,65,72].

Due to divergence, especially in the *gag* and *pol* genes (Figure S3 and Figure 3) between newly reported ALV-like ERV sequences and chicken ASLV, we may conclude that the newly identified ERVs in this paper do not belong to previously reported *Alpharetrovirus* species. We suggest they constitute distant relatives of ASLV due to homology.

## 5. Conclusions

In conclusion, our study highlights the existence of endogenous retroviral sequences in the genomes of at least 12 Galliformes. Their presence is very important, as they may recombine with exogenous or defective alpharetroviruses, generating viruses of unknown pathogenicity. The fact that ORFs deduced from the protein analysis are full-length and preserve the typical protein domains of retroviruses suggests that they may be potentially expressed and complement proteins missing from defective viruses, allowing defective alpharetroviruses to successfully replicate and cause disease. The consistent distribution in five different clades of the ERVs evaluated suggests the stable integration of retroviruses in the germline of the ancestors of the present birds in each of these clades, coevolving with the host and diverging from each other to the point that *pol* analysis supports that each of these clades could constitute a different species and that the taxonomy should be revised. The potential role of these ALV-like ERVs highlights the importance of further studies to better understand the biology of defective viruses, especially in wild Galliformes, their evolution, and the danger they may represent for other wild species and the poultry industry.

## Figures and Tables

**Table 1:** Host and provirus features. Potentially functional ORFs were determined by comparison to retroviral proteins; one species may have several ORFs with premature stop codons, but as long as one did not, that was enough to mark it as functional in this table.

Family	Subfamily	Scientific Name	Species	ERV Structure	Potentially Functional ORFs	ERV Size (Kbp)	Number of Sequences
Odontophoridae		*Callipepla* *californica*	California quail	LTR5′-*gag*-*pol*-*env*-LTR3′	*gag*, *pol*, *env*	7.6	5
Odontophoridae		*Callipepla* *squamata*	Scaled quail	LTR5′-*gag*-*pol*	*gag*, *pol*	5.4 ^1^	4
Odontophoridae		*Colinus* *virginianus*	Virginia quail	?-LTR3′ ^2^		Unknown	1
Phasianidae	Perdicinae	*Alectoris rufa*	Red-legged partridge	LTR5′-*gag*-*pol*-*env*-LTR3′	*gag*, *pol*, *env*	7.4	2
Phasianidae	Perdicinae	*Alectoris magna*	Rusty-necklaced partridge	LTR5′-*gag*-*pol*-*env*-LTR3′	*gag*, *pol*, *env*	7.4	4
Phasianidae	Tetraoninae	*Centrocercus urophasianus*	Greater sage-grouse	LTR5′-*gag*-*pol*-*env*-LTR3′ ^3^	*pol*, *env*	7.7 ^1^	2
Phasianidae	Tetraoninae	*Centrocercus minimus*	Lesser sage-grouse	LTR5′-*gag*-*pol*-*env*-LTR3′ ^3^		7.6 ^1^	2
Phasianidae	Tetraoninae	*Lagopus leucura*	White-tailed ptarmigan	LTR5′-*gag*-*env*-LTR3′		4.8	3
Phasianidae	Tetraoninae	*Lagopus muta*	Rock ptarmigan	LTR5′-*gag-env*-LTR3′	*env*	5.1	2
Phasianidae	Phasianinae	*Phasianus* *colchicus*	Ring-necked pheasant	LTR5′-*gag*-*pol*-*env*-LTR3′	*gag*, *pol*, *env*	7.5	6
Phasianidae	Tetraoninae	*Tympanuchus pallidicinctus*	Lesser prairie chicken	LTR5′-*gag*-*env*-LTR3′		3.9	1
Phasianidae	Tetraoninae	*Tympanuchus cupido*	Greater prairie chicken	LTR5′*-gag-env* ^4^		3.2	1

^1^ Size was calculated by merging ERVs in overlapping regions through alignment. ^2^ *Colinus* ERV structure is unknown. ^3^ The LTR3′ was artificially included in *Centrocercus* ERVs from another ERV since no ERV contains both LTRs. ^4^ *T. cupido* may have a LTR5′; but low quality of the contig prevented checking if it has U5.

## Data Availability

Data can be obtained from the corresponding authors or through the Animal Viruses UCM Research Group, available at: https://www.ucm.es/animalvirucm/ (accessed on 20 November 2023).

## Supplementary Materials

The following supporting information can be downloaded at: https://www.mdpi.com/article/10.3390/microorganisms12010086/s1. Figure S1: Maximum likelihood tree based on LTR5′ DNA sequences; Figure S2: Alignment of LTR sequences; Figure S3: Maximum likelihood tree based on *gag* ORF DNA sequences; Figure S4: Heatmap matrix showing the identity percentages of Env amino acid sequences; Table S1: Genomes searched for the presence of ALV-like ERVs; Table S2: Equivalence between the names we use to refer to ERVs and the contig/scaffold in which they are located; Table S3: GenBank sequences used in this study; Table S4: Identities between LTR5′ and LTR3′ of all ERVs with both LTRs identified in this study; Table S5: ORFs of a protein size and C-terminus similar to ALV-E.
