# Supplementary material for: Comparison of Endogenous Alpharetroviruses (ALV-like) across Galliform Species: New Distant Proviruses
Source: Microorganisms. 2023 Dec 31;12(1):86. doi: 10.3390/microorganisms12010086 (PMC10820513; doi:10.3390/microorganisms12010086)
Supplement: Supplementary file 1 [file microorganisms-12-00086-s001.zip › microorganisms-2766067-supplementary.pdf]

Supplementary material

Table S1: Genomes searched for the presence of ALV-like ERVs. *Species in which we were able to locate recognizable ERVs.

Order	Family	Common name	Species	Accession number (NCBI assembly)
Anseriformes	Anatidae	Mallard	*Anas platyrhynchos*	GCA_008746955
Anseriformes	Anatidae	Canada goose	*Branta canadensis*	GCA_006130075
Anseriformes	Anatidae	Black swan	*Cygnus atratus*	GCF_013377495
Anseriformes	Anatidae	Mute swan	*Cygnus olor*	GCA_009769485
Columbiformes	Columbidae	Rock pigeon	*Columba livia*	GCF_000337935
Coraciiformes	Meropidae	Carmine bee eater	*Merops nubicus*	GCA_009819595
Cuculiformes	Cuculidae	Common cuckoo	*Cuculus canorus*	GCA_017976305
Galliformes	Numididae	Helmeted guineafowl	*Numida meleagris*	GCF_002078875
Galliformes	Odontophoridae	California quail	*Callipepla californica**	GCA_023055725
Galliformes	Odontophoridae	Scaled quail	*Callipepla squamata**	GCA_002218305
Galliformes	Odontophoridae	Northern bobwhite	*Colinus virginianus**	GCA_008692595
Galliformes	Phasianidae	Przewalski's partridge	*Alectoris magna**	GCA_030625065
Galliformes	Phasianidae	Red-legged partridge	*Alectoris rufa**	GCA_019345075
Galliformes	Phasianidae	Gunnison sage-grouse	*Centrocercus minimus**	GCA_005890655
Galliformes	Phasianidae	Greater sage-grouse	*Centrocercus urophasianus**	GCF_019232065
Galliformes	Phasianidae	Golden pheasant	*Chrysolophus pictus*	GCA_003413605
Galliformes	Phasianidae	Japanese quail	*Coturnix japonica*	GCF_001577835
Galliformes	Phasianidae	Brown eared pheasant	*Crossoptilon mantchuricum*	GCA_019593555
Galliformes	Phasianidae	White-tailed ptarmigan	*Lagopus leucura**	GCF_019238085
Galliformes	Phasianidae	Rock ptarmigan	*Lagopus muta**	GCF_023343835
Galliformes	Phasianidae	Turkey	*Meleagris gallopavo*	GCA_905368555
Galliformes	Phasianidae	Green peafowl	*Pavo muticus*	GCA_015227815
Galliformes	Phasianidae	Ring-necked pheasant	*Phasianus colchicus**	GCF_004143745

Galliformes	Phasianidae	Greater prairie chicken	*Tympanuchus cupido**	GCA_001870855
Galliformes	Phasianidae	Lesser prairie chicken	*Tympanuchus pallidicinctus**	GCF_026119805
Passeriformes	Acanthisittidae	Rifleman	*Acanthisitta chloris*	GCA_016880875
Passeriformes	Corvidae	American crow	*Corvus Brachyrhynchos*	GCF_000691975
Passeriformes	Fringillidae	House finch	*Haemorhous mexicanus*	GCA_027406555
Passeriformes	Hirundinidae	Barn swallow	*Hirundo rustica*	GCA_015227815
Passeriformes	Motacillidae	White wagtail	*Motacilla alba*	GCF_015832195
Passeriformes	Muscicapidae	Collared flycatcher	*Ficedula albicollis*	GCF_000247815
Passeriformes	Paradisaeidae	Obi Paradise crow	*Lycocorax pyrrhopterus*	GCA_014706295
Passeriformes	Paridae	Blue tit	*Cyanistes caeruleus*	GCF_002901205
Passeriformes	Paridae	Great tit	*Parus major*	GCF_001522545
Passeriformes	Parulidae	Common yellowthroat	*Geothlypis trichas*	GCA_009764545
Passeriformes	Passerellidae	Song sparrow	*Melospiza melodia*	GCA_022749775
Passeriformes	Passeridae	Eurasian tree sparrow	*Passer montanus*	GCF_014805655
Passeriformes	Strildidae	Common canary	*Serinus canaria*	GCF_007115625
Passeriformes	Strildidae	Zebra finch	*Taeniopygia guttata*	GCA_003957525
Passeriformes	Sturnidae	Common starling	*Sturnus vulgaris*	GCF_001447265
Passeriformes	Sylviidae	Wrentit	*Chamaea fasciata*	GCA_029207785
Passeriformes	Sylviidae	Blackcap	*Sylvia atricapilla*	GCA_009819655
Passeriformes	Thraupidae	Small tree finch	*Camarhynchus parvulus*	GCF_901933205
Passeriformes	Thraupidae	Medium ground finch	*Geospiza fortis*	GCF_000277835
Passeriformes	Turdidae	Swainson's thrush	*Catharus ustulatus*	GCA_009819505
Passeriformes	Turdidae	European robin	*Erithacus rubecula*	GCA_903797595
Passeriformes	Tyrannidae	Willow flycatcher	*Empidonax traillii*	GCF_003031625

Phoenicopteriformes	Phoenicopteridae	American flamingo	*Phoenicopterus ruber*	GCA_009819775
Psittaciformes	Psittacidae	Monk parakeet	*Myiopsitta monachus*	GCA_017639245
Psittaciformes	Psittacidae	Grey parrot	*Psittacus erithacus*	GCA_009867235
Sphenisciformes	Spheniscidae	Emperor penguin	*Aptenodytes forsteri*	GCF_000699145
Strigiformes	Tytonidae	Barn owl	*Tyto alba*	GCF_018691265
Struthioniformes	Struthionidae	African ostrich	*Struthio camelus*	GCF_000698965

Table S2: Equivalence between the names we use to refer to ERVs and the contig/scaffold in which they are located. ERV starting position as well as the DNA strand is also included. ERV sequences found were named according to the host species, followed by numbers corresponding to the contigs from lowest to highest ID number in each host species. If there were several ERVs in the same contig, a second number was added using the position from the 5′ start of the contig as criterion.

Subject Name (Code)	Sequence ID	ERV start position	Strand	Type
Alectoris-rufa1	CAMZON010000074	25424403	-1	WGS contig
Alectoris-rufa2	JADBKV010009649	7020	-1	WGS contig
Alectoris-magna1	CM060560	189936656	-1	Genome
Alectoris-magna2-1	CM060571	20365772	-1	Genome
Alectoris-magna2-2	CM060571	20422673	-1	Genome
Alectoris-magna3	CM060577	10044346	1	Genome
Callipepla-californica1	JALIRH010000007	13610422	-1	WGS contig
Callipepla-californica2	JALIRI010000093	1118264	1	WGS contig
Callipepla-californica3	JALIRH010000158	311730	1	WGS contig
Callipepla-californica4	JALIRI010000160	638155	1	WGS contig
Callipepla-californica5	JALIRI010000324	80173	-1	WGS contig
Callipepla-squamata1	MCFN01000256	1079060	1	WGS contig
Callipepla-squamata2	MCFN01002646	87	1	WGS contig
Callipepla-squamata3	MCFN01004905	5735	-1	WGS contig
Callipepla-squamata4	MCFN01005269	6372	-1	WGS contig
Centrocercus-urophasianus1	JAHKSY010000017	3352724	-1	WGS contig
Centrocercus-urophasianus2	JAHKSY010000020	2560960	1	WGS contig
Centrocercus-minimus1	SPOS01000018	1863703	-1	WGS contig
Centrocercus-minimus2	SPOS01000084	404824	1	WGS contig
Colinus-virginianus1	VONY02000001	11768068	1	WGS contig
Lagopus-leucura1-1	JAHKMA010000015	35675795	1	WGS contig
Lagopus-leucura1-2	JAHKMA010000015	28225527	1	WGS contig
Lagopus-leucura2	JAHKMA010000085	54414	1	WGS contig
Lagopus-muta1	JAMCCT010000006	131386259	1	WGS contig
Lagopus-muta2	JAMCCT010000013	67496142	-1	WGS contig
Phasianus-colchicus1-1	WUCP01000001	41775392	1	WGS contig
Phasianus-colchicus1-2	WUCP01000001	59007839	1	WGS contig
Phasianus-colchicus1-3	WUCP01000001	41760765	1	WGS contig
Phasianus-colchicus2	WUCP01000003	70547314	1	WGS contig
Phasianus-colchicus3	WUCP01000011	205230	1	WGS contig
Phasianus-colchicus4	WUCP01000018	3706230	-1	WGS contig
Tympanuchus-pallidicinctus1	JAPCYR010000034	720334	1	WGS contig
Tympanuchus-cupido1	MOXI01000063	659153	1	WGS contig

Table S3: GenBank sequences used in this study.

Name	Type	NCBI accession number	Length (bp)
ALV-A	Exogenous	DQ365814	7704
ALV-B	Exogenous	JF826241	7747
RSV-C	Exogenous	J02342	9625
RSV-D	Exogenous	D10652	9317
ALV-E (phylogenies)	Endogenous	KY235336	9679
ALV-E (data mining)	Endogenous	AY013303	7525
ALV-F (env CDS)	Endogenous[1]	AY608692	1887
ALV-J	Exogenous	Z46390	7841
ALV-K	Exogenous	KY581580	7703
Avian carcinoma Mill virus	Exogenous defective	M14008	2626
Avian sarcoma CT10 virus	Exogenous defective	NC038922	2428
Fujinami sarcoma virus	Exogenous defective	AF033810	4540
UR2 sarcoma virus	Exogenous defective	M10455	3166
Y73 sarcoma virus	Exogenous defective	L21974	5184
LPDV	Exogenous	KC802224	7432
LPDV (partial)	Exogenous	U09568	7143
Bonasa umbellus ERV (TERV)	Endogenous	AF289082	3698

[1]ALV-F *env* originates from an endogenous retrovirus; however, sequence AY608692 was most likely acquired from sequencing a recombinant exogenous virus carrying subgroup F *env* gene. ERV, endogenous retrovirus. LPDV, lymphoproliferative disease virus. TERV, tetraonine endogenous retrovirus.

Table S4:Identities between LTR5′ and LTR3′ of ERVs identified in this study. The size of the LTR ranged from 250 to 304 bp.

ERV name	Identity between LTRs
Alectoris-magna1	250/251=0.996
Alectoris-magna2-1	249/251=0.992
Alectoris-magna2-2	250/251=0.996
Alectoris-magna3	251/251=1
Alectoris-rufa1	250/251=0.996
Callipepla-californica1	303/303=1
Callipepla-californica4	304/304=1
Callipepla-californica5	304/304=1
Lagopus-leucura1-1	376/376=1
Phasianus-colchicus1-1	250/251=0.996
Phasianus-colchicus1-2	251/251=1
Phasianus-colchicus1-3	248/251=0.988
Phasianus-colchicus2	250/250=1
Phasianus-colchicus3	251/251=1
Phasianus-colchicus4	252/252=1
Lagopus-muta1	302/302=1
Lagopus-muta2	302/302=1
Tympanuchus-pallidicinctus1	302/302=1

Table S5: ORFs of a protein size and C-terminus similar to ALV-E, without early stop codons in ERVs, and thus considered to be functional, as in ALV-E. It was not possible to study *Colinus* ORFs. Length in amino acids is written in each cell.

ERV	*gag*	*pol*	*env*
Alectoris-rufa1			573
Alectoris-rufa2	736	895	
Alectoris-magna1	731	895	573
Alectoris-magna2-1	731	895	573
Alectoris-magna2-2	731	895	573
Alectoris-magna3		895	
Callipepla-californica1	711	901	605
Callipepla-californica2	711	901	
Callipepla-californica3	711	901	605
Callipepla-californica4		901	605
Callipepla-californica5	711	901	605
Callipepla-squamata1	711		
Callipepla-squamata2	705	899	
Callipepla-squamata3			
Callipepla-squamata4	709		
Centrocercus-urophasianus1		895	
Centrocercus-urophasianus2		895	571
Centrocercus-minimus1			
Centrocercus-minimus2			
Colinus-virginianus1	?	?	?
Lagopus-leucura1-1			
Lagopus-leucura1-2			
Lagopus-leucura2			
Lagopus-muta1			
Lagopus-muta2			568
Phasianus-colchicus1-1	709	895	628
Phasianus-colchicus1-2		895	628
Phasianus-colchicus1-3	709	895	
Phasianus-colchicus2	709	895	628
Phasianus-colchicus3	709	895	628
Phasianus-colchicus4	710	895	628
Tympanuchus-pallidicinctus1			
Tympanuchus-cupido1			
Total number	16	19	15

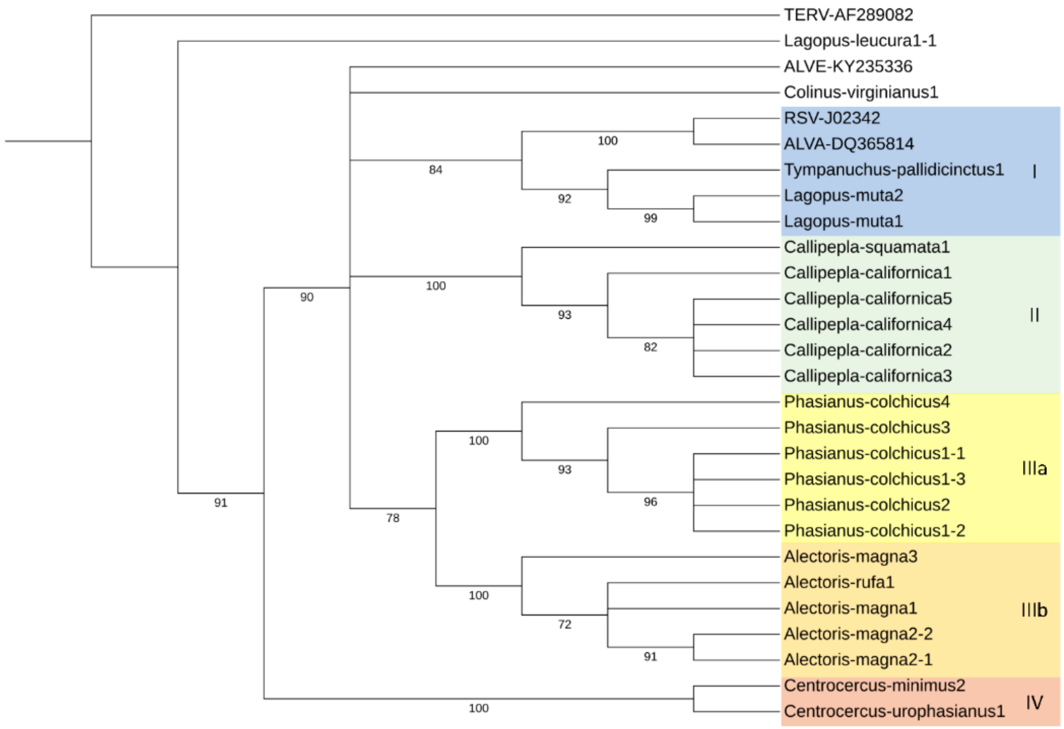

Figure S1: Maximum likelihood tree based on LTR5′ DNA sequences (389 bp) of several proviruses detected in twelve species of Galliformes, using K2+I method (bootstrap value = 1000 replicates).

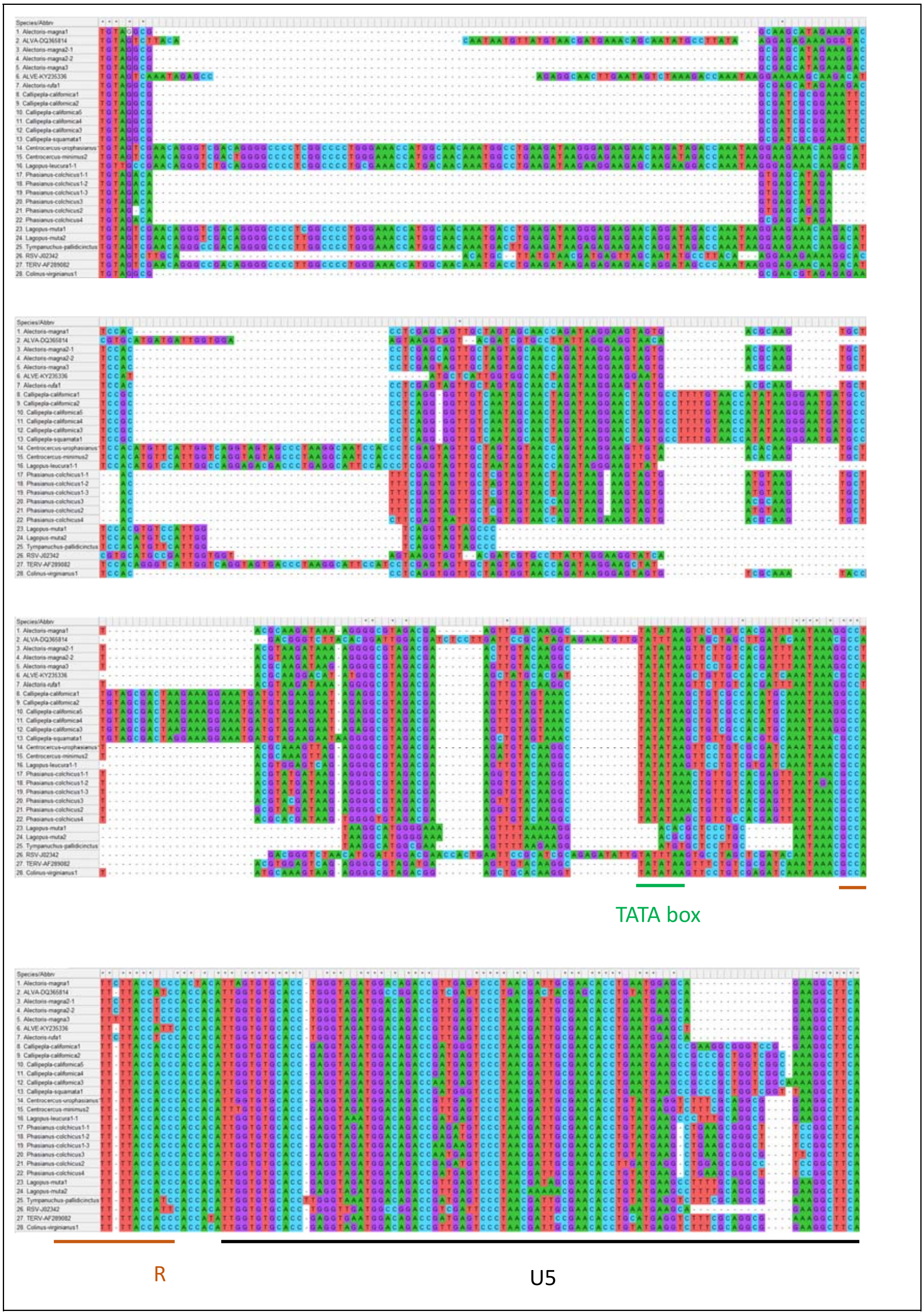

Figure S2: Alignment of LTR sequences. Accession numbers are shown in Table S2 and Table S3. The U3 region is highly divergent among the newly discovered ERVs until it reaches the point of the TATA box (TATATAA); but R-U5 is mostly conserved. ALV-A, ALV-E, TERV and RSV sequences are included for comparison.

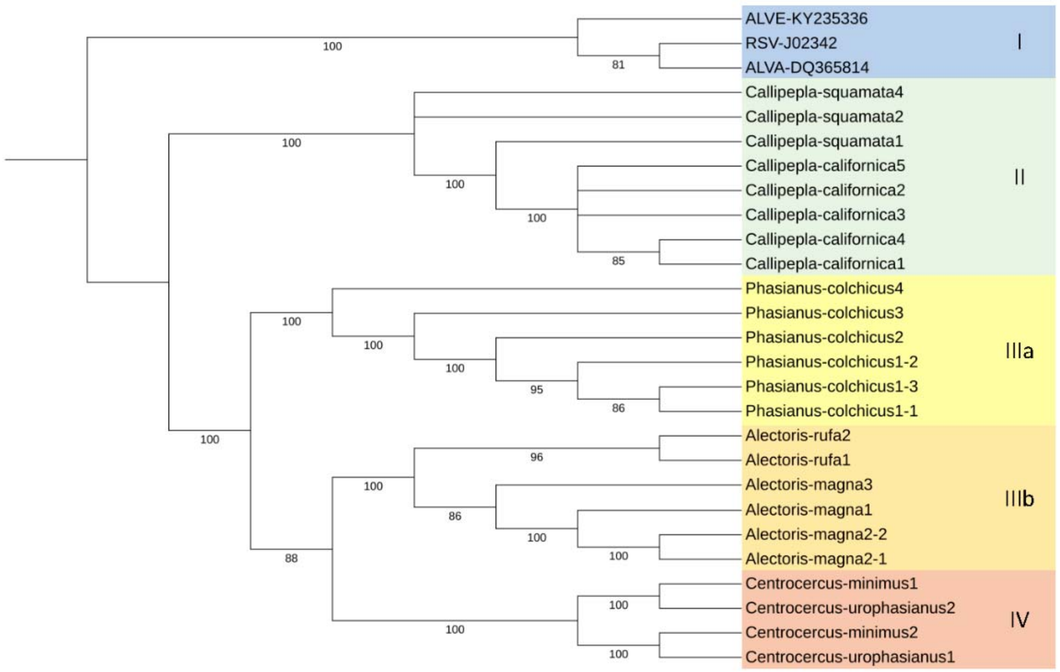

Figure S3: Maximum likelihood tree based on *gag* ORF DNA sequences (2211 bp) of several proviruses detected in seven species of Galliformes, using T92+G method (bootstrap value = 1000 replicates).

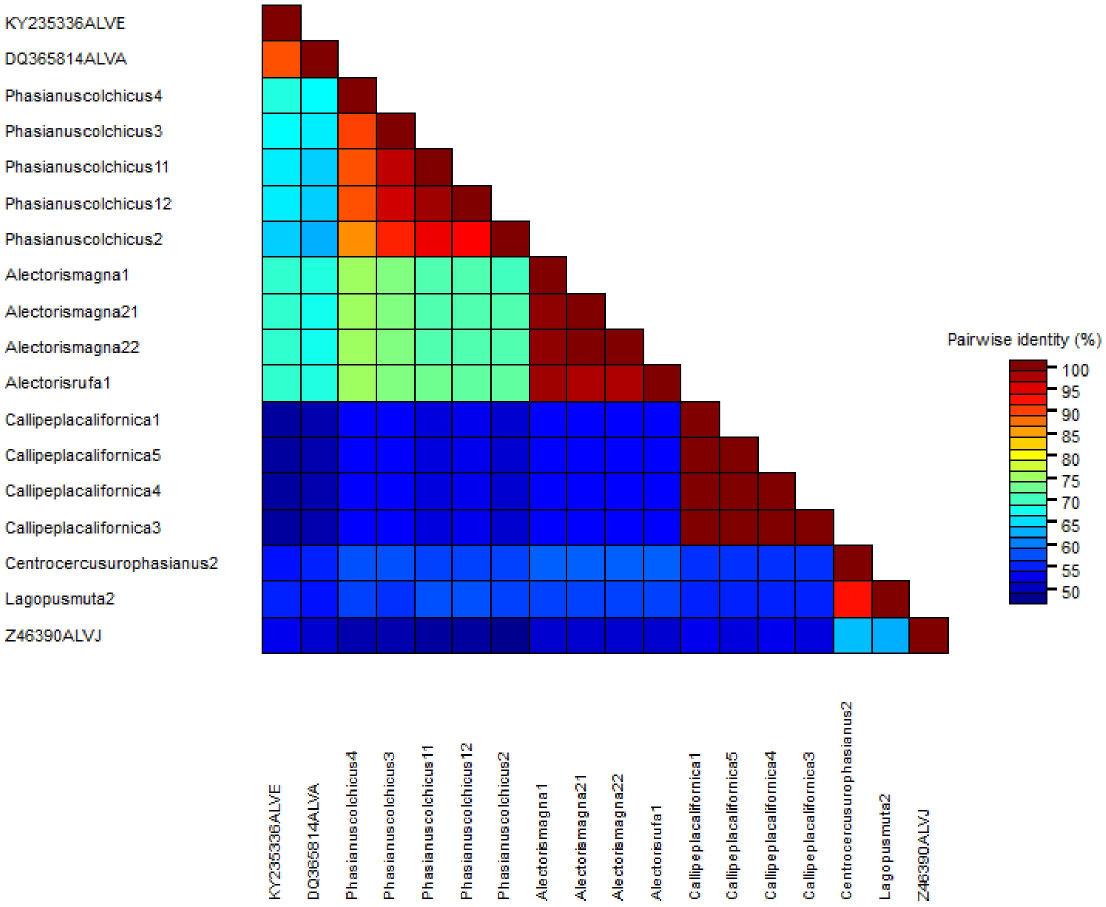

Figure S4: Heatmap matrix showing the identity percentages of Env aminoacid sequences. It has been created using sequence demarcation tool 1.2.
